# The *BVES* Gene Regulates the Homeostasis of Deer Antler Mesenchymal Stem Cells Through Wnt Signaling

**DOI:** 10.3390/biology14091210

**Published:** 2025-09-07

**Authors:** Hong Chen, Chuan Lin, Xin Xiang, Chenchen Yang, Chunmei Han, Qinghua Gao

**Affiliations:** 1 College of Life Science and Technology, Tarim University, Alar 843300, China; chenhong090588@163.com; 2 College of Animal Science and Technology, Tarim University, Alar 843300, China; linchuan0528@163.com (C.L.); xiang991201@163.com (X.X.); chen2yang0218@163.com (C.Y.); 3 Key Laboratory of Tarim Animal Husbandry Science and Technology, Xinjiang Production and Construction Corps, Alar 843300, China; 4 Key Laboratory of Livestock and Forage Resources Utilization Around Tarim, Ministry of Agriculture and Rural Areas, Alar 843300, China

**Keywords:** BVES, antler mesenchymal stem cells, Wnt signaling pathway, proliferation capacity

## Abstract

(1) Background: The proliferation and differentiation of antler mesenchymal stem cells (MSCs) are vital for antler growth. Our group found that BVES is downregulated during peak antler growth but is highly expressed in mesenchymal tissues, suggesting its role in regulating antler homeostasis. (2) Methods: We manipulated BVES expression in antler MSCs using lentiviral plasmids and siRNA, and then assessed its effects on MSC proliferation via CCK-8 and EdU assays, while also analyzing changes in Wnt pathway gene expression. (3) Results: Overexpression of BVES significantly decreased the CCK-8 OD value of antler MSCs (*p* < 0.05), and EdU assays revealed a significantly lower cell positivity rate compared to the control group (*p* < 0.01). In contrast, suppression of BVES resulted in a significant increase in the OD value (*p* < 0.05) and a significantly higher cell positivity rate than the control group (*p* < 0.05). Additionally, overexpression of BVES significantly downregulated the expression of key Wnt signaling pathway genes (*LRP6*, *DVL3*, *β-catenin*, *c-Myc*), while suppression of BVES led to their upregulation. (4) Conclusions: In summary, the *BVES* gene inhibits the proliferation of antler MSCs by suppressing the Wnt signaling pathway. It may serve as a key gene in maintaining antler MSC homeostasis and regulating antler growth, providing a new perspective for understanding the mechanisms of antler growth.

## Simple Summary

Antlers are the only mammalian organs capable of complete regeneration. They grow at an extremely rapid rate yet rarely undergo cancerous transformation. This rapid and controlled proliferation relies on the precise regulation of antler MSCs, but the mechanisms underlying the maintenance of their homeostasis remain unclear. This study investigates the effect of BVES on the proliferative capacity of antler MSCs and its regulatory mechanism. Through experiments involving the overexpression and interference of BVES, we confirmed that BVES inhibits the proliferation of antler MSCs by suppressing the activity of key molecules (LRP6/β-catenin/c-Myc) in the Wnt/β-catenin pathway. This study reveals a new mechanism underlying the cancer-free regeneration of deer antlers, providing a theoretical basis for mammalian tissue regeneration.

## 1. Introduction

The Tarim red deer (*Cervus elaphus yarkandensis*), a unique subspecies of red deer that has evolved in the desert environment of Xinjiang, is renowned for its rapidly growing antlers. Studies indicate that the daily growth rate of red deer antlers during their peak growth phase can reach up to 2.75 cm [1]. This remarkable growth, driven by the proliferation and differentiation of antler MSCs, exhibits a unique biological feature: despite resembling tumor growth rates, it maintains ordered tissue structure without cancerous transformation. Such controlled proliferation makes antlers an ideal model for studying mammalian tissue regeneration, which holds significant implications for regenerative medicine and tumor suppression.

Antler growth primarily relies on the apical growth center, where pedicle periosteum cells (PPCs) differentiate into reserve mesenchymal cells (RMCs, i.e., antler MSCs). These MSCs proliferate and differentiate into cartilage and bone tissues, driving rapid antler growth. However, the key factors regulating the proliferative homeostasis of antler MSCs, ensuring rapid proliferation without malignant transformation, remain unclear.

BVES (blood vessel epicardial substance) was first identified by Reese et al. [2,3] in chick heart tissue and was subsequently isolated by Andre et al. [4]. It is a member of the Popeye domain-containing (POPDC) gene family, exhibiting only 25% sequence homology with the family members POPDC2 and POPDC3. The protein structure comprises an extracellular amino terminus, three transmembrane domains, and an intracellular carboxy terminus. Parang et al. [5] reported that the human BVES transmembrane protein contains an extracellular structural domain of 42 amino acids, which includes two N-glycosylation sites. Its carboxy-terminal region harbors a highly conserved Popeye structural domain, along with a carboxy-terminal domain (CTD) adjacent to it [4,6]. The Popeye domain-containing protein consists of 94 amino acids and serves as a critical site for cAMP binding [4,5]. The CTD, also composed of 94 amino acid residues, has the ability to interact with several proteins, including ZO-1 and GEFT [5], which are essential for regulating the biological activity of BVES proteins [5,7].

Current research has found that BVES can participate in the regulation of cell proliferation and homeostasis by modulating cell junctions and signaling pathways. In cancer research, BVES is often silenced in malignant tumors (e.g., lung and colon cancers) due to promoter hypermethylation; its re-expression inhibits cancer cell proliferation and attenuates Wnt signaling [8,9,10]. Jayagopal et al. [11] demonstrated that overexpression of BVES in uveal melanoma cells also diminished the proliferative capacity of these tumor cells. Conversely, the downregulation of BVES expression in liver tumors facilitates the invasion and metastasis of liver cancer cells while enhancing their proliferation. In intestinal crypt stem cells, BVES knockout enhances Wnt activity and promotes excessive stem cell proliferation [12], suggesting that BVES may regulate stem cell homeostasis via the Wnt pathway. Whether this mechanism applies to the cancer-free proliferation of antler MSCs remains unexplored.

Antler growth is an exceptionally complex process. Although the growth rate of antler tissue resembles that of a tumor, it consistently maintains an orderly tissue structure without becoming cancerous. Consequently, the relationship between the growth and development mechanisms of antlers and the malignant proliferation of cancer cells has become a prominent topic of research. The Wnt/β-catenin signaling pathway plays a crucial role in various physiological processes, including cell proliferation, differentiation, apoptosis, migration, invasion, and tissue homeostasis [13,14]. Increasing evidence suggests that overactive Wnt/β-catenin signaling is associated with the development of malignant tumors [15,16].

Our previous study found that BVES is significantly downregulated during the peak antler growth period and highly expressed in mesenchymal tissues [17], a spatio-temporal pattern closely associated with antler MSCs proliferation. The Wnt/β-catenin pathway is known to play a crucial role in cell homeostasis [13,14], but whether BVES interacts with this pathway to regulate antler MSC proliferation remains unknown. Here, we manipulated BVES expression in antler MSCs to investigate its effects on proliferation and explore its regulatory relationship with the Wnt signaling pathway, aiming to elucidate the molecular mechanisms maintaining antler MSCs homeostasis.

## 2. Materials and Methods

### 2.1. Materials

Tarim red deer antler tissues were collected from the deer farm of the Thirty-first Group, Second Division, Xinjiang Production, and Construction Corps. The maral deer was anesthetized with xylazine hydrochloride injection (1.0 mL/100 kg). The surface of the antler velvet was washed with sodium bicarbonate solution, and then the antler was sawed off. The antler was immersed in 75% Ethanol Solution (SUN, ZhuYuan Reagent Co., Ltd., Tianjing, China) for 10 s, repeated three times, and then soaked in PBS solution (Servicebio, Wuhan, China) for 20 s, repeated three times. Finally, the antler was immersed in PBS and transported back to the laboratory.

### 2.2. Methods

#### 2.2.1. Expression of the *BVES* Gene in Antler Tissue at Different Growth Stages

Total RNA was extracted from antler tissues at 30, 53, and 80 days using the Trizol method. The quality and concentration of the RNA samples were evaluated through 1% agarose gel electrophoresis. Samples exhibiting an OD260/280 ratio between 1.8 and 2.0 were reverse-transcribed into cDNA using HyperScript III RT SuperMix (EnzyArtisan, Shanghai, China) and subsequently stored at −80 °C. Specific primers were designed based on the predicted mRNA sequence of the deer *BVES* gene (XM_043890183.1) obtained from GeneBank, utilizing Primer 6.0 software (refer to Table 1). The relative expression levels of the *BVES* gene were quantified using the 2 × S6 Universal SYBR qPCR Mix (EnzyArtisan, Shanghai, China) and a real-time fluorescence qPCR system (LongGene, Hangzhou, China), following the manufacturer’s instructions.

#### 2.2.2. Cloning of the CDS Region of the *BVES* Gene from the Antler of the Tarim Red Deer and Construction of an Overexpression Vector

The amplification of the CDS region was performed using the BVES-1 sequence, as outlined in Table 1, which served as the cloning primer for this region. The amplified target fragment was subsequently purified and recovered, followed by ligation into the pMD19-T plasmid. After sequencing verification, the recombinant plasmid pMD19-T-BVES was extracted and sent to Hanheng Biotechnology Co., Ltd., Shanghai, China, for lentivirus packaging. During the lentivirus packaging process, the transfer plasmid carrying the target gene, the packaging plasmid providing viral structural proteins, and the envelope plasmid supplying envelope proteins were prepared. Co-transfection was then performed using HEK293T cells. The pre-warmed fresh medium (Gibco Life Sciences, Waltham, MA, USA) was replaced 24 h post-transfection, and the cell supernatant was collected at 48 and 72 h, respectively. Following this, centrifugation was carried out to remove cell debris, and the virus was concentrated. The viral titer was subsequently detected using fluorescence microscopy (Nikon T1-SM, CHANSN, Shanghai, China), quantitative PCR (qPCR), or ELISA [18,19]. Finally, the virus was stored at −80 °C for subsequent infection experiments on deer antler MSCs.

#### 2.2.3. BVES Protein Bioinformatics Analysis

By predicting and analyzing the BVES protein domain through the NCBI (https://www.ncbi.nlm.nih.gov/Structure/cdd/wrpsb.cgi, accessed on 7 February 2025) and PFAM databases (https://www.ebi.ac.uk/interpro/entry/pfam/#table, accessed on 20 February 2025), as well as utilizing the online tool InterProScan 105.0 (https://www.ebi.ac.uk/interpro/, accessed on 28 April 2025), we compared the conservation of amino acid sequences in the Popeye domain of BVES proteins among the Tibetan antelope, human, and mouse using SnapGene 6.0.2.

#### 2.2.4. Isolation of Deer Antler MSCs

In the laminar flow hood, the 53d antler was longitudinally sectioned. Following the descriptions provided by Price et al. [20], Li et al. [21], and Kuzmova et al. [22], the milky white, soft tissue layer beneath the velvet skin was identified; this layer caps the underlying cartilaginous tissue. Utilizing the method described by Chen et al. [23], this tissue layer was carefully isolated. In accordance with the optimized culture method for antler MSCs developed by Wang [24], the freshly separated interstitial tissue from the Tarim maral deer antler was thoroughly washed with PBS and subsequently minced into 1 mm^2^ pieces using ophthalmic scissors. The minced tissue was then digested with trypsin (Beyotime Biotechnology, Shanghai, China) until individual cells were separated. The isolated cells were subsequently transferred to culture flasks for primary cell culture. Multiple studies have demonstrated that the antler mesenchymal layer is predominantly composed of MSCs, which possess the ability for continuous proliferation and the potential for multidirectional differentiation [25,26,27,28]. In our preliminary cell cultures, we observed that these cells exhibit the same functional characteristics as previously studied antler MSCs, along with elevated expression levels of the *c-Myc* gene [24,29]. Furthermore, regarding cell morphology and structure, they align with the antler MSCs cultured by Chen et al. [23]. The cells are primarily spindle-shaped, displaying regular morphology and an orderly arrangement pattern. The cell nuclei are relatively large, and the nucleoli are distinctly visible. Consequently, we can conclude that the cultured cells are indeed antler MSCs.

#### 2.2.5. BVES Overexpressing Lentivirally Transfected Antler MSCs

The Tarim red deer antler MSCs were cultured to passage P3 and subsequently transfected according to the instructions provided by Hanheng Biotechnology Co., Ltd. At 48 h post-transfection, RT-qPCR was performed, and at 72 h post-transfection, Western blotting was used to assess the overexpression efficiency. Once a significant increase in BVES expression was confirmed, subsequent experiments were conducted.

#### 2.2.6. Transfection of Antler MSCs by siRNA

In this experiment, three pairs of siRNAs constructed by Hanheng Biotechnology Co., Ltd., Shanghai, China, were utilized to conduct in vitro transfection experiments on antler MSCs. After 48 and 72 h of transfection, the interference efficiency was assessed using RT-qPCR and Western blot analysis, leading to the selection of the optimal siRNA2 (Sense strand: GAUUUGUUCAGAAGAUUAATT; Antisense strand: UUAAUCUUCUGAACAAAUCTT) for subsequent experiments.

#### 2.2.7. Assay for Cell Count

Cell proliferation ability was assessed using Enhanced Cell Counting Kit-8 (Beyotime Biotechnology, Shanghai, China). A total of 5 × 10^3^ cells were seeded into 96-well plates and transfected with either BVES-overexpressing lentivirus or siRNA once cell confluence reached 80%. Subsequently, 10 μL of sterile CCK-8 reagent was added to each well at 6, 12, 24, 48, 72, 96, and 120 h of incubation. After a 2 h incubation period, the absorbance at 450 nm was measured using a multimode microplate reader (KAIAO, Beijing, China) to construct a growth curve for antler MSCs. (The Control group served as the negative control, while the Lenti-vector group acted as the positive control for overexpression. The Lenti-BVES group was designated as the experimental group for overexpression. The si-NC group functioned as the positive control for interference, and the siRNA2 group was identified as the experimental group for interference).

#### 2.2.8. Cell Proliferation Capacity Assay

The proliferative capacity of antler MSCs was assessed using the EdU kit (Beyotime Biotechnology, Shanghai, China). A total of 3 × 10^4^ cells were seeded into 48-well plates and transfected with either BVES-overexpressing lentivirus or siRNA once they reached 80% confluence. The original medium was replaced with pre-warmed fresh medium 24 h post-transfection, and incubation was continued for an additional 48 h, during which 500 µL of EdU was added for 3 h. Subsequently, the cells were fixed with 4% paraformaldehyde (Biosharp, Hefei, China) at room temperature and permeabilized with 0.3% Triton X-100 (Beyotime Biotechnology, Shanghai, China). Finally, the cells were incubated in 100 µL of Click reaction mixture for 30 min and then treated with 100 µL of Hoechst 33342 (5 µg/mL) for 10 min. Images were captured using fluorescence microscopy (Nikon T1-SM, CHANSN, Shanghai, China). The percentage of EdU-positive cells was calculated by dividing the number of EdU-positive cells by the total number of Hoechst-stained cells (the experimental grouping was the same as in Section 2.2.8).

#### 2.2.9. Detection of Genes Related to the Wnt Pathway

Specific primers designed using Primer 6.0. *GAPDH* (NM_001034034.2) served as the internal reference gene, while key Wnt pathway genes included Cervus elaphus LRP6 (XM_043880589), Cervus elaphus DVL3 (XM_043875513), Cervus elaphus β-catenin (XM_043886767.1), and Cervus elaphus c-Myc (XM_043880136) (see Table 1). RT-qPCR was conducted following the protocol provided by the 2 × S6 Universal SYBR RT-qPCR Mix (EnzyArtisan) kit, with the final results calculated using the relative expression method, specifically the 2^−ΔΔCt^ method.

### 2.3. Statistical Analysis

Statistical analyses were conducted using SPSS version 23 and GraphPad Prism version 5. Normally distributed measures are expressed as mean ± standard deviation (x ± s). Significance of differences between two groups was assessed using the T-test, while comparisons of means among three or more groups were analyzed using one-way ANOVA. Asterisks denote significance levels, with “ns” indicating *p* > 0.05, “*” indicating *p* < 0.05, and “**” indicating *p* < 0.01.

## 3. Results

### 3.1. Expression of the BVES Gene in Antler Tissue at Different Growth Stages

**Figure 1:**
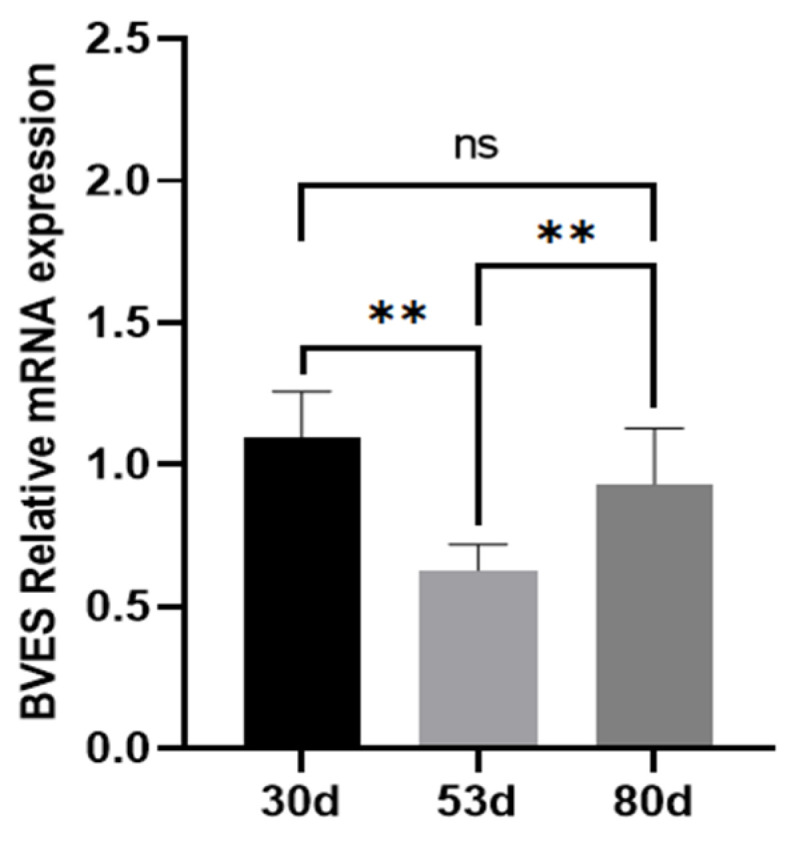
Relative expression levels of the *BVES* gene in antler tissue at different growth stages. Note: Experimental unit is deer, and *n* = 3 for each stage. “ns” indicates *p* > 0.05, “**” indicates *p* < 0.01.

RT-qPCR analysis demonstrated that the *BVES* gene was expressed in the antler tissue of Tarim red deer throughout all three growth stages. The expression level of the *BVES* gene was significantly downregulated on day 53 of antler growth (*p* < 0.01) and significantly upregulated on day 80 (*p* < 0.01). However, no significant difference in *BVES* gene expression was observed between day 30 and day 80 of antler growth (*p* > 0.05) (see Figure 1).

### 3.2. Construction and Protein Structure Prediction of the BVES Overexpression Vector in Tarim Red Deer Antler

**Figure 2:**
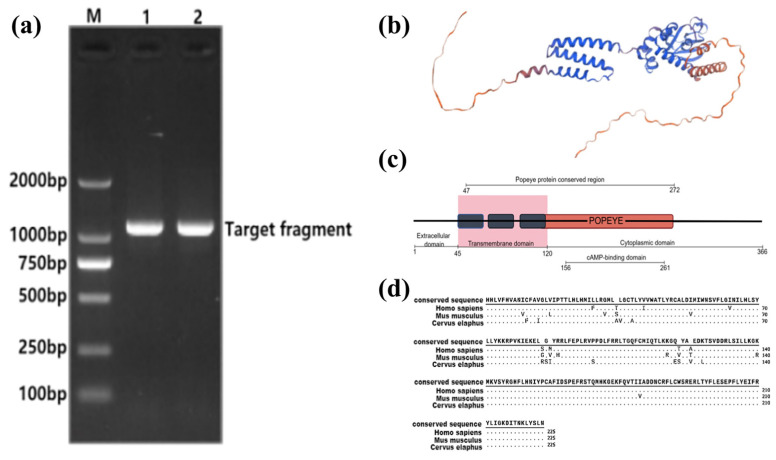
Cloning of the BVES coding sequence from Tarim red deer and prediction of its protein structure. (**a**) Bacterial liquid PCR identification. Lane M is the marker; Lanes 1 and 2 are PCR products of positive colonies; (**b**) Predicted tertiary structure of BVES protein of Tarim red deer antler. (**c**) Schematic representation of the structure of the BVES protein in Tarim red deer; the dark blue in the figure represents the transmembrane domain, and the red represents the POPEYE domain. (By Figdraw 2.0). (**d**) Sequence comparison of the conserved region of the Popeye structural domain of the Tarim red deer BVES protein.

The cloning process resulted in a 1098 bp coding sequence (CDS) of the *BVES* gene derived from the antler of the Tarim red deer, encoding a total of 366 amino acids (Figure 2a).

The extracellular structural domain of the BVES protein comprises 45 amino acids, with its transmembrane structural domain situated between amino acid positions 46 and 119, and the cytoplasmic structural domain spanning positions 120 to 366 (Figure 2b). The amino acid positions 47 to 272 correspond to the conserved region of the Popeye structural domain, which has been documented in both the NCBI and PFAM databases (PFAM04831). The intracytoplasmic Popeye structural domain consists of 152 amino acids, with the binding site for cAMP located within positions 156 to 261 of the amino acid sequence [30] (Figure 2c). Comparative analysis revealed that the amino acid sequence of the conserved region of the Popeye structural domain exhibits a similarity of 92.89% and 90.67% to that of human (NP_001186492.1, PFAM PF04831) and mouse (NP_077247.1, PFAM PF04831), respectively (Figure 2d).

### 3.3. Effect of BVES Overexpression on the Proliferative Capacity of Antler MSCs

**Figure 3:**
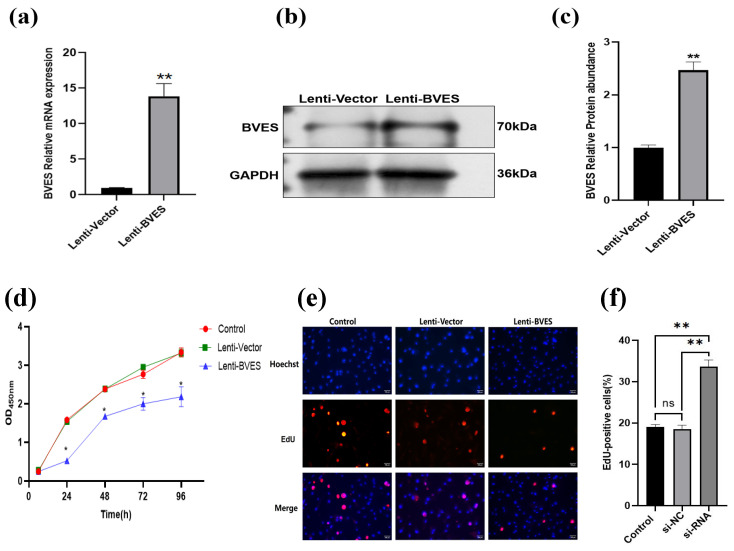
The impact of BVES overexpression on the proliferation of antler MSCs. (**a**) Expression of *BVES* gene mRNA in antler MSCs 48 h after lentiviral transfection. (**b**) Expression of BVES protein in antler MSCs 72 h after lentiviral transfection. (**c**) Analysis of BVES protein overexpression results. (**d**) Viability of antler MSCs detected by CCK8 assay. The CCK8 detection experiments with overexpression and interfering of BVES were conducted simultaneously, using the same control. (**e**) Staining result graph of the proliferation of antler MSCs detected by the EdU method. (**f**) EdU positivity rate. Note: The original Western blot images are presented in Figure S1 in the Supplementary Materials. EdU-positive cells (red) and Hoechst-stained cells (blue). Double-stained cells represent EdU-positive cells. Experimental unit is antler MSCs, and *n* = 3 for each group experiment. In all charts with error bars, the data represent the mean ± standard deviation, “ns” indicates *p* > 0.05, “*” indicates *p* < 0.05, and “**” indicates *p* < 0.01.

As illustrated in Figure 3a, the relative expression level of the *BVES* gene in passage P3 antler MSCs transfected with an overexpression lentivirus was significantly elevated compared to that of the control group (*p* < 0.01). Furthermore, as demonstrated in Figure 3b,c, the results from Western blotting revealed that the protein expression level of BVES in the BVES overexpression group was markedly higher than that in the control group (*p* < 0.01). These findings are consistent with the RT-qPCR results, thereby confirming the successful overexpression of BVES.

The results of adding CCK-8 to passage P3 antler MSCs indicated a significant reduction in the proliferative capacity at 24, 48, 72, and 96 h following BVES in the Lenti-BVES group compared to the control and Lenti-vector groups (*p* < 0.05). No significant differences were observed among the three groups during the first 24 h (*p* > 0.05) (Figure 3d).

Additionally, EdU results demonstrated that the percentage of positive cells in the Lenti-BVES group (6.10 ± 0.90%) was significantly lower than that in the Lenti-vector group (18.43 ± 0.58%) and the control group (22.43 ± 0.54%). There was no significant difference between the Lenti-vector group and the control group (*p* > 0.05). These findings are consistent with the CCK-8 cell proliferation assay results, suggesting that the overexpression of BVES inhibits the proliferative capacity of antler MSCs (Figure 3e,f).

### 3.4. The Effect of Interfering with BVES on the Proliferation Ability of Deer Antler MSCs

**Figure 4:**
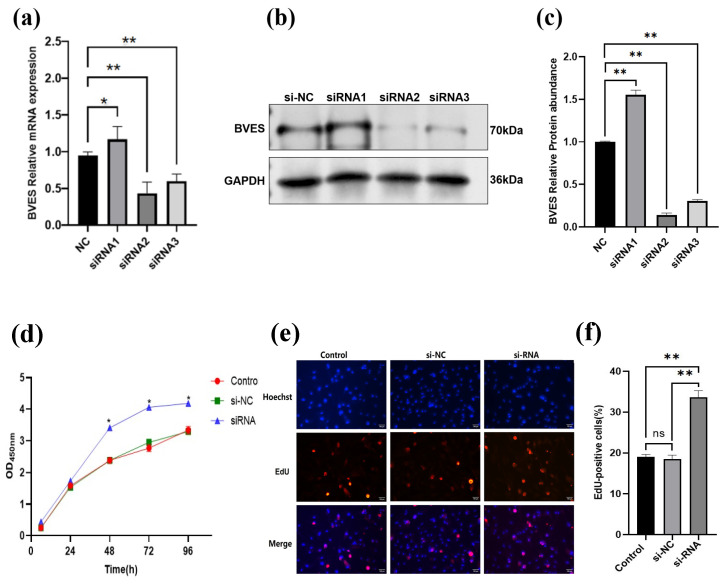
The Effect of interfering with *BVES* on the proliferation ability of deer antler MSCs. (**a**) Expression of *BVES* gene mRNA in antler mesenchymal stem cells 48 h after transfection with different siRNAs. (**b**) Expression of BVES protein in antler mesenchymal stem cells 72 h after transfection with different siRNAs. (**c**) Analysis of BVES protein interference results. (**d**) Viability of antler MSCs detected by CCK8 assay. The CCK8 detection experiments with overexpression and interfering of BVES were conducted simultaneously, using the same control. (**e**) Staining result graph of the proliferation of antler MSCs detected by the EdU method. (**f**) EdU positivity rate. Note: The original Western blot images are shown in Figure S2 in the Supplementary Materials. EdU-positive cells (red) and Hoechst-stained cells (blue). Double-stained cells represent EdU-positive cells. Experimental unit is antler MSCs, and *n* = 3 for each group experiment. In all charts with error bars, the data represent the mean ± standard deviation, “ns” indicates *p* > 0.05, “*” indicates *p* < 0.05, and “**” indicates *p* < 0.01.

As illustrated in Figure 4a, after transfection of passage P3 antler mesenchymal stem cells with different siRNAs, the expression level of BVES in the siRNA1-infected group was significantly elevated compared to that in the negative control (NC) group (*p* < 0.05). Conversely, the expression levels of BVES in both the siRNA2 and siRNA3 groups were extremely significantly reduced relative to the NC group (*p* < 0.01). Notably, the interference efficiency of siRNA2 surpassed that of siRNA3, establishing siRNA2 as the optimal candidate for RNA interference. Furthermore, as depicted in Figure 4b,c, both siRNA2 and siRNA3 effectively suppressed the expression of BVES protein, aligning with the observed trend in the relative expression of the *BVES* gene. Consequently, siRNA2 was chosen for the subsequent interference experiments.

The results of adding CCK-8 to passage P3 antler MSCs indicated a significant increase in their proliferative capacity (*p* < 0.05) compared to the control and si-NC groups at 48, 72, and 96 h following BVES interference. No significant differences were observed among the three groups during the initial 48 h (*p* > 0.05) (Figure 4d).

Furthermore, the EdU assay results demonstrated that the positive cell rate in the siRNA group (33.60 ± 0.9%) was significantly higher than that in the si-NC group (18.43 ± 0.58%) and the control group (19 ± 0.35%). Additionally, there was no significant difference between the si-NC group and the control group (*p* > 0.05). These findings are consistent with the results of the CCK-8 cell proliferation assay, suggesting that the inhibition of the *BVES* gene enhances the proliferative capacity of antler MSCs (Figure 4e,f).

### 3.5. Effect of the BVES Gene on Wnt Signaling in Deer Antler MSCs Proliferation

**Figure 5:**
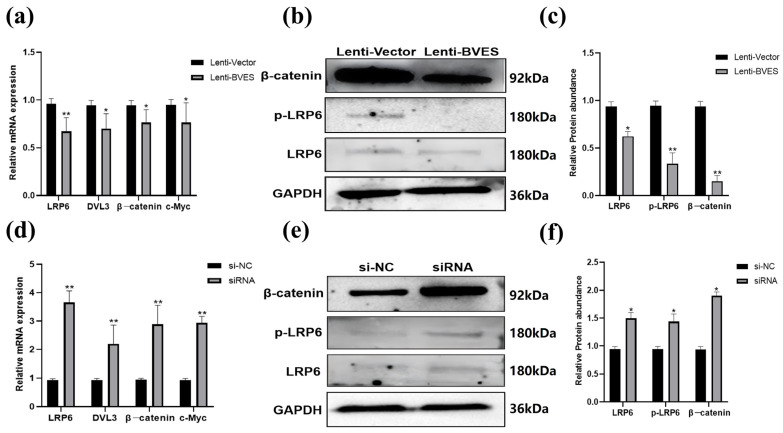
Effect of the *BVES* gene on Wnt signaling in deer antler MSCs proliferation. (**a**) Expression of key genes in the Wnt signaling pathway after overexpression of the *BVES* gene; (**b**) Western blot detection of LRP6, pLRP6 and β-catenin protein expression in the Wnt pathway after BVES overexpression; (**c**) Quantification of the expression of each protein. (**d**) Expression of key genes in the Wnt pathway after inhibition with the *BVES* gene; (**e**) Western blot detection of protein expression of LRP6, pLRP6, and β-catenin in the Wnt pathway after inhibition with BVES; (**f**) Quantification of expression of each protein. Note: The original Western blot images are shown in Figures S3–S5 in the Supplementary Materials. Experimental unit is antler MSCs, and *n* = 3 for each group experiment. “*” indicates *p* < 0.05, and “**” indicates *p* < 0.01.

After overexpression of BVES, the mRNA expression levels of the *LRP6*, *DVL3*, *β-catenin*, and *c-Myc* genes in the Wnt pathway were assessed using RT-qPCR. The results indicated that the expression of these genes was significantly lower in the BVES overexpression group compared to the Lenti-vector group. Additionally, Western blot analysis revealed that the protein levels of LRP6, pLRP6, and β-catenin were all significantly decreased following BVES overexpression (*p* < 0.05) (Figure 5a–c).

After Interfering with *BVES*, the mRNA expression levels of *LRP6*, *DVL3*, *β-catenin*, and *c-Myc* genes in the Wnt signaling pathway were significantly elevated following interference with the *BVES* gene compared to the si-NC group (*p* < 0.01). Additionally, Western blot analysis revealed that the protein levels of LRP6, pLRP6, and β-catenin were also significantly increased after interference (*p* < 0.05) (Figure 5d–f).

## 4. Discussion

Preliminary studies conducted by our research group indicate that the expression of the *BVES* gene in deer antler mesenchymal tissue is significantly higher than in other tissues [17]. Notably, the expression of this gene was downregulated during the peak growth period of the antler, while it was highly significantly upregulated during the late growth period. These findings tentatively suggest that the *BVES* gene may play a regulatory role in the growth and development of antler MSCs.

In recent years, a growing body of evidence has indicated that BVES possesses significant potential in inhibiting tumor invasion and metastasis. Shi et al. [31] demonstrated that overexpression of BVES in gallbladder cancer cells significantly inhibited the proliferation and invasion capabilities of these tumor cells. Similarly, Jayagopal et al. [11] reported that BVES overexpression in uveal melanoma cells attenuated their proliferative ability. Conversely, interference with BVES expression in liver tumors promoted the invasion and metastasis of hepatocellular carcinoma cells and enhanced their proliferative capacity. Furthermore, Reddy et al. [12] found that knocking out the *BVES* gene in mice resulted in enhanced proliferation of intestinal crypt stem cells, alongside increased activity of the Wnt signaling pathway. This suggests that the *BVES* gene plays a crucial role in regulating the activity of intestinal stem cells and the Wnt signaling pathway. In this study, we investigated the effects of BVES on antler MSCs and obtained results consistent with those observed in tumor cells and small intestinal crypt stem cells. Overexpression of BVES significantly inhibited the proliferative capacity of antler MSCs, while downregulating the mRNA expression and protein levels of LRP6, β-catenin, and c-Myc within the Wnt signaling pathway. Conversely, inhibition of BVES significantly enhanced the proliferative capacity of antler MSCs and notably increased the expression of LRP6, DVL3, β-catenin, and *c-Myc* genes, along with their corresponding protein levels within the Wnt signaling pathway.

The inhibitory effects of BVES on the Wnt/β-catenin pathway are evident across various tissues, indicating that its function is deeply conserved among vertebrates. However, as the only mammalian organ capable of complete regeneration, the regulation of BVES in deer antlers exhibits unique spatiotemporal dynamics. The periodic regeneration of deer antlers may drive the “reversible inhibition” of BVES function. During the peak growth phase (45–65 d), the downregulation of BVES facilitates explosive proliferation, while its upregulation in the later stages (70–95 d) terminates growth and initiates differentiation. This regulatory pattern may stem from the selective adaptation of deer antlers to rapid tissue regeneration. Whether similar BVES-Wnt regulatory modules exist in other regeneration models, such as salamander limbs, remains an open question. If such modules do not exist, it may suggest that the antler-specific regulation of BVES represents an innovative mechanism for mammalian regeneration.

The Wnt/β-catenin signaling pathway is a multifunctional signaling pathway that plays a crucial role in embryonic development, cell proliferation, and differentiation, and is integral to the regulation of various cells and tissues. Within this pathway, the phosphorylation of LRP6 is essential for the transduction of classical Wnt signals. Specifically, the phosphorylation of LRP6 facilitates the entry of β-catenin into the nucleus, thereby promoting the activation of the Wnt signaling pathway, which serves as a critical link in this signaling cascade [32]. Mount et al. [33] investigated the role of the classical Wnt signaling pathway in different tissues of antlers using immunohistochemistry and found that the expression of β-catenin protein was markedly low in cartilaginous and osteogenic tissues. In contrast, the expression was significantly higher in undifferentiated mesenchymal tissues, suggesting for the first time that the Wnt/β-catenin signaling pathway plays a vital role in regulating mesenchymal cell proliferation and bone formation in antlers. Furthermore, a recent study [34] demonstrated that BVES can modulate Wnt signaling by decreasing the levels of the LRP6 receptor and β-catenin in colon cancer cells. The findings indicated that overexpression of BVES resulted in the downregulation of LRP6 expression and a reduction in its phosphorylation level, leading to the attenuation of Wnt signaling, while the downregulation of BVES produced the opposite effect.

The *c-Myc* gene is a target of the Wnt signaling pathway. Han [29] demonstrated that the *c-Myc* gene is involved in the proliferation and differentiation of antler skin during the rapid growth period of antlers. The present experiment further confirms that the *c-Myc* gene also promotes the proliferation of antler MSCs. Parang et al. [35] observed enhanced Wnt signaling and increased c-Myc levels following BVES knockdown in mouse tumor cells. Conversely, BVES overexpression reduced the stability of c-Myc and increased its ubiquitin-mediated degradation. Through a yeast two-hybrid (Y2H) screen, they identified that the regulatory subunit of protein phosphatase 2A (PP2A), PR61α, interacts with BVES and enhances PP2A activity, which is crucial for the degradation of c-Myc. This interaction was identified as a mechanism by which BVES regulates intracellular c-Myc levels. These findings are consistent with the results obtained by Parang et al. [35] in mouse tumor cells.

At the structural level, the intricate membrane systems within the cell precisely compartmentalize functional regions through elements such as transmembrane domains and nuclear localization signals. Consequently, proteins are directed to the endoplasmic reticulum–Golgi apparatus during translation for post-translational modifications, and are rapidly anchored to their sites of action [36,37]. In this study, the full-length CDS cloned from Tarim red deer antler encodes a transmembrane BVES protein that lacks a signal peptide and contains three transmembrane domains (46–119 aa), an extracellular segment of 45 aa, and a cytoplasmic segment of 247 aa. Residues 47–272 constitute the highly conserved Popeye domain, which shares 92.89% and 90.67% sequence identity with human and mouse orthologues, respectively. AlphaFold2 predictions suggest that residues 156–261 within the Popeye domain may harbor a cAMP-binding site, although experimental validation is still required. Given this putative cAMP-binding capacity of the Popeye domain, we speculate that a local rise in cAMP during the late growth phase might facilitate the interaction between BVES and the PP2A regulatory subunit PR61α, thereby accelerating c-Myc degradation and attenuating Wnt signaling to help terminate rapid antler growth. This hypothesis remains to be experimentally confirmed.

**Figure 6:**
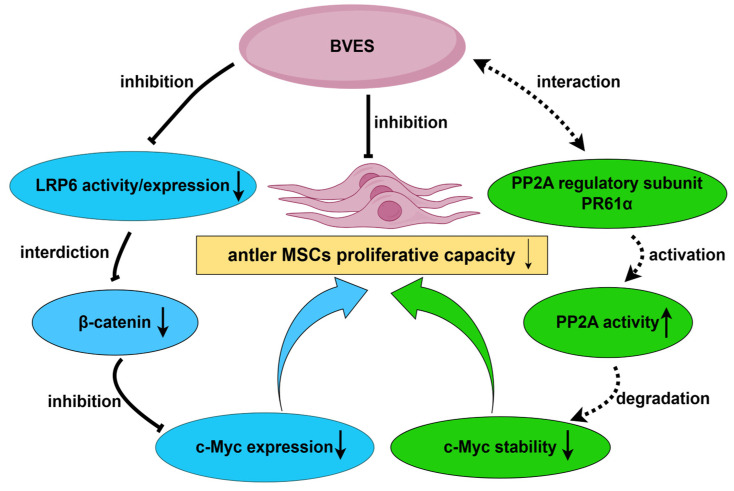
Mechanistic diagram of BVES-mediated regulation of antler MSC proliferative capacity. BVES can reduce the intracellular accumulation of β-catenin by inhibiting the activity of LRP6, thereby suppressing the expression of c-Myc and ultimately inhibiting the proliferation ability of antler MSCs. In addition, BVES may activate PP2A through its interaction with the PP2A regulatory subunit PR61α, thereby reducing the stability of c-Myc, accelerating its degradation, and ultimately leading to a decrease in the proliferation ability of antler MSCs. Note: Solid arrows denote signaling pathways already validated in antler mesenchymal cells; dashed arrows denote potential pathways that remain to be tested. This image was drawn by Figdraw 2.0.

In summary, during the proliferation of antler MSCs, BVES can influence the Wnt signaling pathway by modulating the activity of LRP6, which in turn reduces the expression of downstream target genes (Figure 6). Additionally, the *BVES* gene may directly regulate the expression level of the *c-Myc* gene by interacting with the regulatory subunit PR61α of PP2A, thereby affecting the growth homeostasis of antler MSCs. This intricate regulatory mechanism necessitates further experimental validation. This study reveals that BVES functions as a “molecular brake” in deer antler MSCs, maintaining the balance between regeneration and differentiation by regulating the Wnt signaling pathway and the stability of c-Myc at multiple levels. This mechanism not only shares conserved pathways associated with tumor suppression but also exhibits unique regulatory plasticity. It provides a theoretical foundation for a deeper understanding of the growth mechanisms of deer antlers and serves as a significant supplement to the theory of deer antler growth homeostasis, while also offering new insights into mammalian regeneration and disease.

## 5. Conclusions

The proliferative capacity of antler MSCs is a crucial factor for antler growth, development, and regeneration. The results of this study indicate that the proliferative capacity of antler MSCs decreases following BVES overexpression, accompanied by downregulation of key genes in the Wnt signaling pathway, including LRP6, DVL3, β-catenin, and c-Myc. Conversely, inhibition of BVES leads to an increase in the proliferative capacity of antler MSCs, with upregulation of the same Wnt signaling pathway genes. Thus, it is posited that BVES inhibits the proliferation of antler MSCs by suppressing the Wnt signaling pathway during the peak growth period of antlers. This gene may play a pivotal role in maintaining the rapid growth of antlers without leading to cancerous transformations. These novel findings are expected to provide significant theoretical insights into the mechanisms underlying the rapid growth of antlers.

## Figures and Tables

**Table 1:** Amplification primer information.

Primer Name	Primer Sequence	Annealing Temperature	Fragment Length
BVES-1	F: ATGCGAGGAATTTTCAAGATGAATTATACT	63	1098
	R: AGGCCACTGACGAACTTTAAATGTATTTG		
BVES	F: TGACGACCGTCTGAGTATTCTCCTG	61	133
	R: TCACCTTTGTGCATCTGGGTTGATC		
LRP6	F: CCATCCGCCGCTCCTTCATAG	58	127
	R: TCAGTGCCAGTGTCTGTCCAATAG		
DVL3	F: GAGCACCATCACCTCCACCAGCTCC	61	161
	R: TGAGCCACATTCGGTCACGGACCTCT		
β-catenin	F: TGCTGAAGGTGCTGTCTGTCTG	58	133
	R: TTCCGTAGAGTCCAAAGGCAGTTC		
c-Myc	F: CAAATGTGCCAGCCCAAGGTTTTC	65	130
	R: CTCTGGGATCTGGTCACGAAGAGCA		
GAPDH	F: TGTTTGTGATGGGCGTGAACCA	55	154
	R: ATGGCGTGGACAGTGGTCATAA		

Note: BVES-1 is a primer for cloning the CDS region.

## Data Availability

The original contributions presented in the study are included in the article; further inquiries can be directed to the corresponding authors.

## Supplementary Materials

The following supporting information can be downloaded at https://www.mdpi.com/article/10.3390/biology14091210/s1. Figure S1. Results of BVES protein overexpression. Figure S2. Interference effects of different siRNA groups on the BVES protein. Figure S3. Expression of Wnt signaling-related proteins after overexpression of BVES. Figure S4. Expression of Wnt signaling-related proteins after overexpression of BVES. Figure S5. Expression of Wnt signaling-related proteins after interference of BVES.
