# Supplementary material for: The BVES Gene Regulates the Homeostasis of Deer Antler Mesenchymal Stem Cells Through Wnt Signaling
Source: Biology (Basel). 2025 Sep 7;14(9):1210. doi: 10.3390/biology14091210 (PMC12467380; doi:10.3390/biology14091210)
Supplement: Supplementary file 1 [file biology-14-01210-s001.zip › biology-3697800-supplementary.pdf]

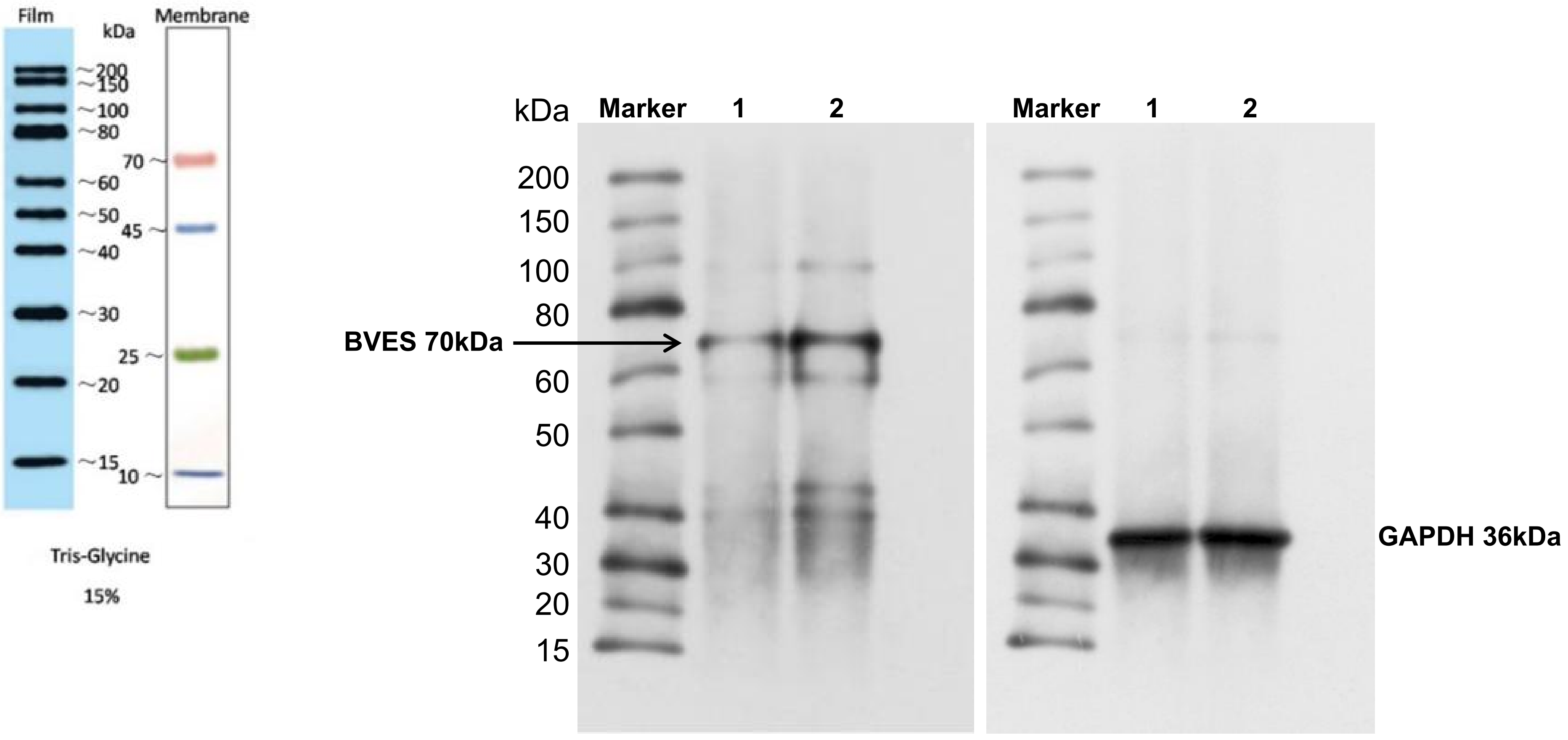

Figure S1. Results of BVES protein overexpression.

Note: Lanes 1 is Lenti-Vector; Lanes 2 is Lenti-BVES (Expose for 15 seconds).

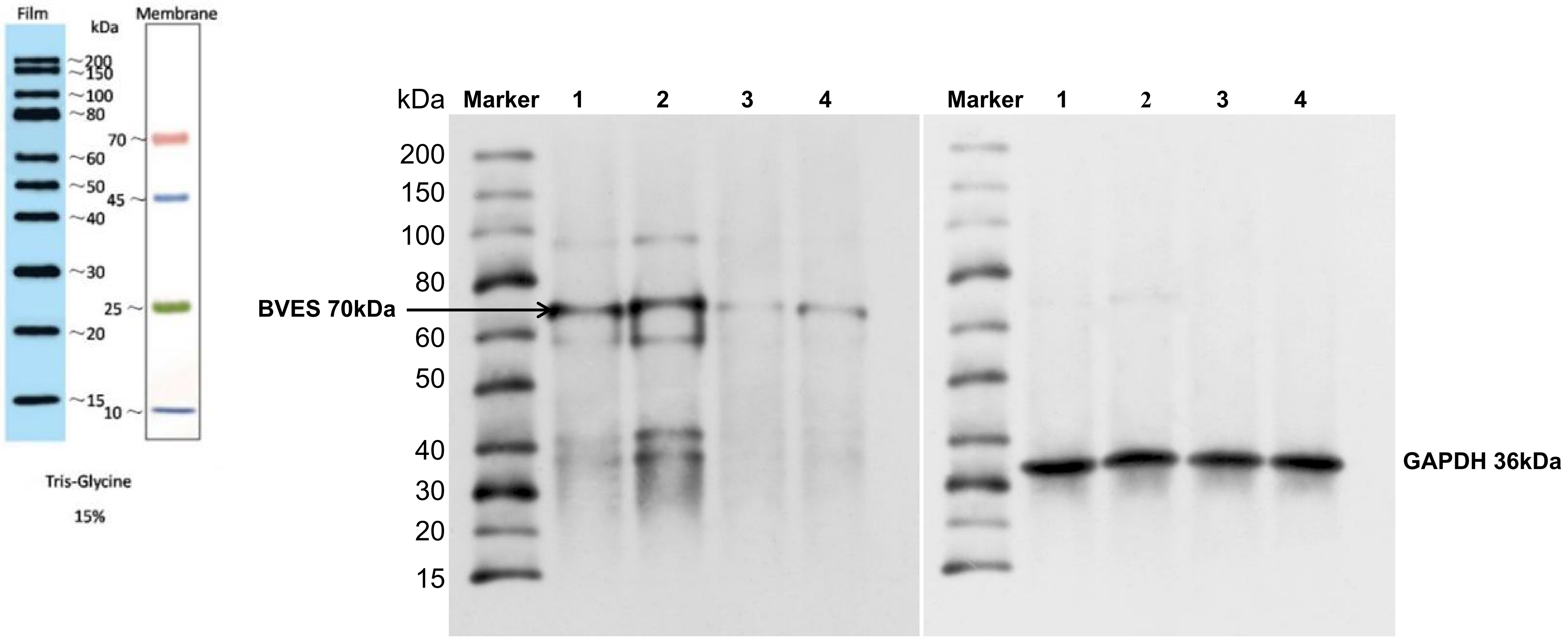

Figure S2. Interference effects of different siRNA groups on the BVES protein

Note: Lanes 1 is si-NC; Lanes 2 is siRNA1; Lanes 3 is siRNA2 and Lanes 4 is siRNA3 (Expose for 15seconds).

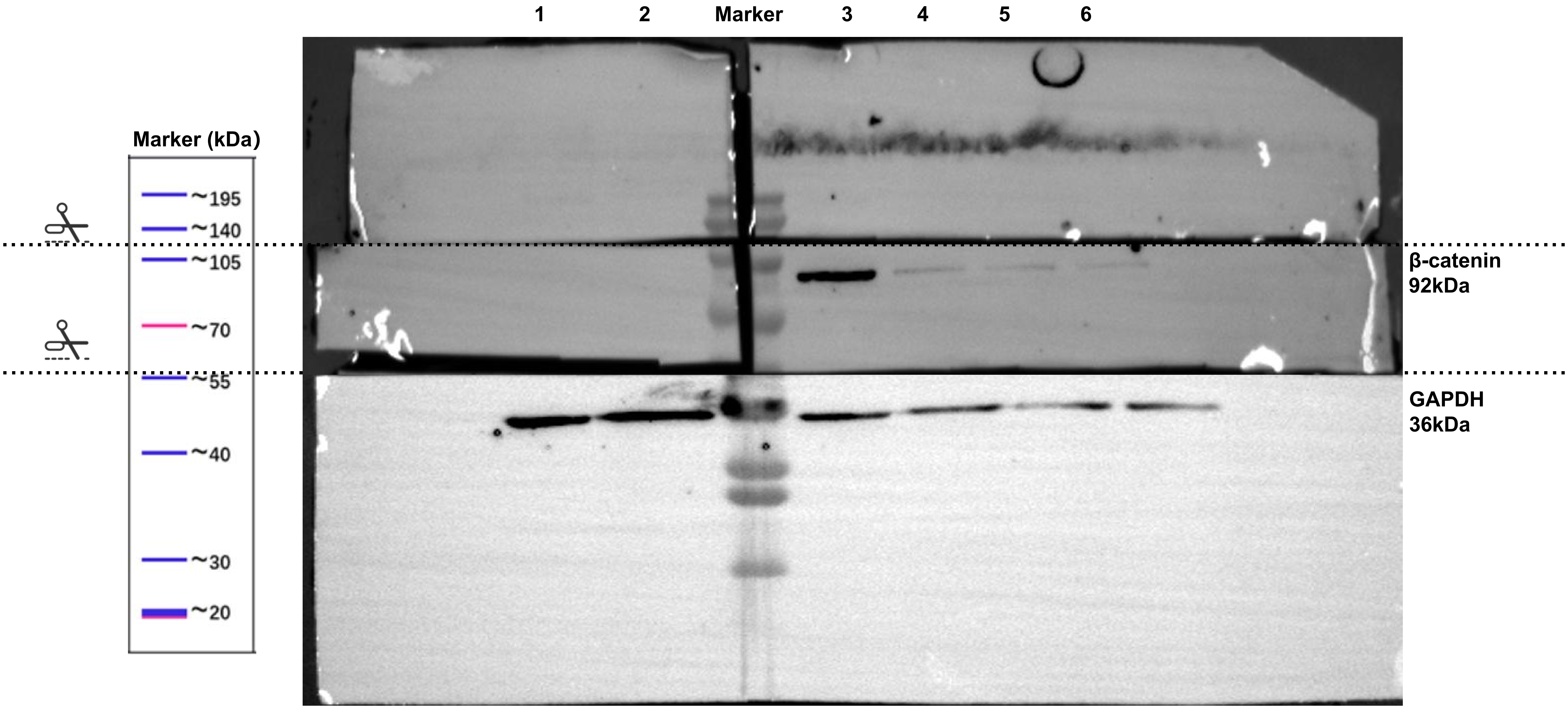

Figure S3. Expression of Wnt signaling-related proteins after overexpression of BVES

Note: Lanes 1 and 3 are both Lenti-Vector; Lanes 2, 4, 5, and 6 are all Lenti-BVES (Expose for 10 seconds).

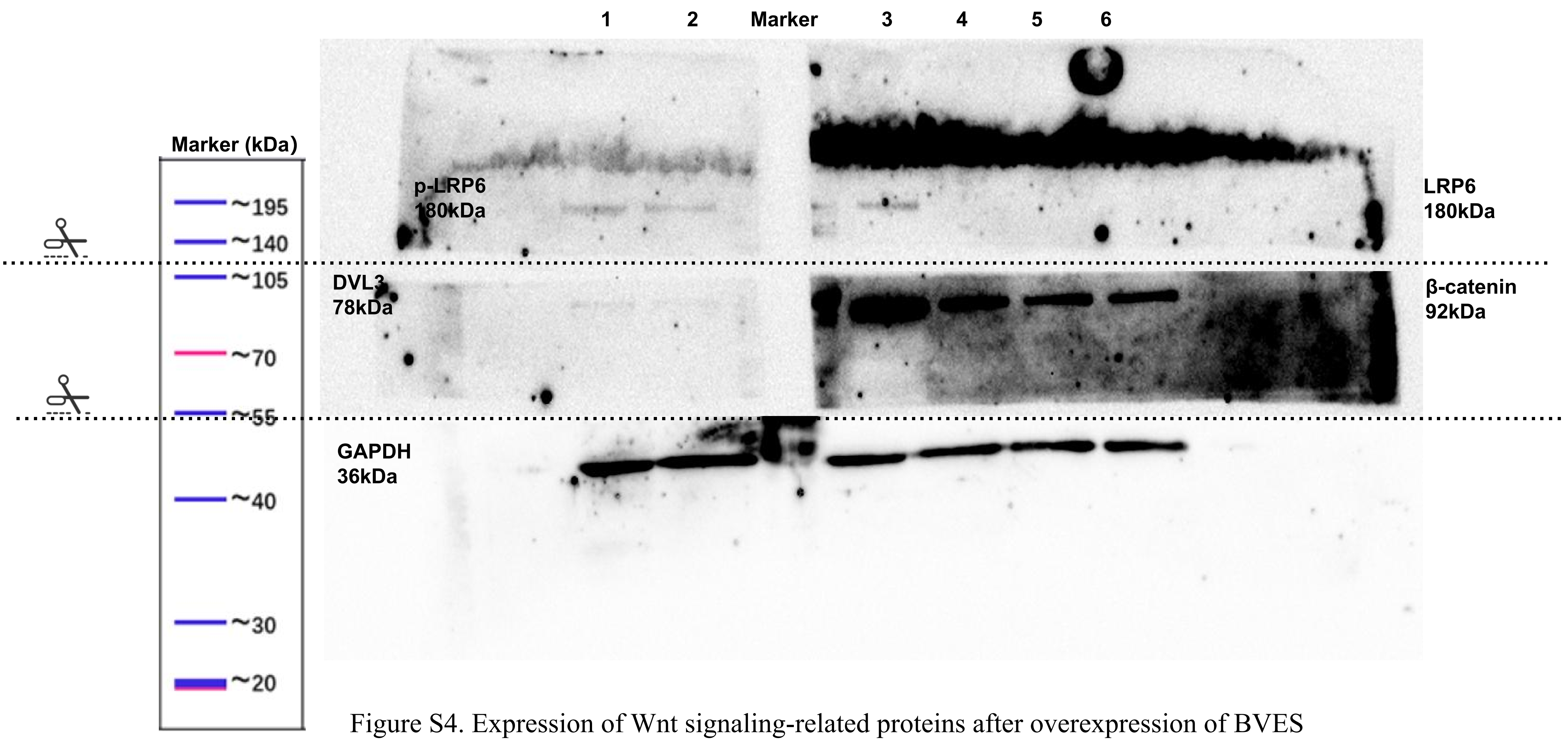

Figure S4. Expression of Wnt signaling-related proteins after overexpression of BVES

Note: Lanes 1 and 3 are both Lenti-Vector; Lanes 2, 4, 5, and 6 are all Lenti-BVES (Expose for Expose for 2 minutes.).

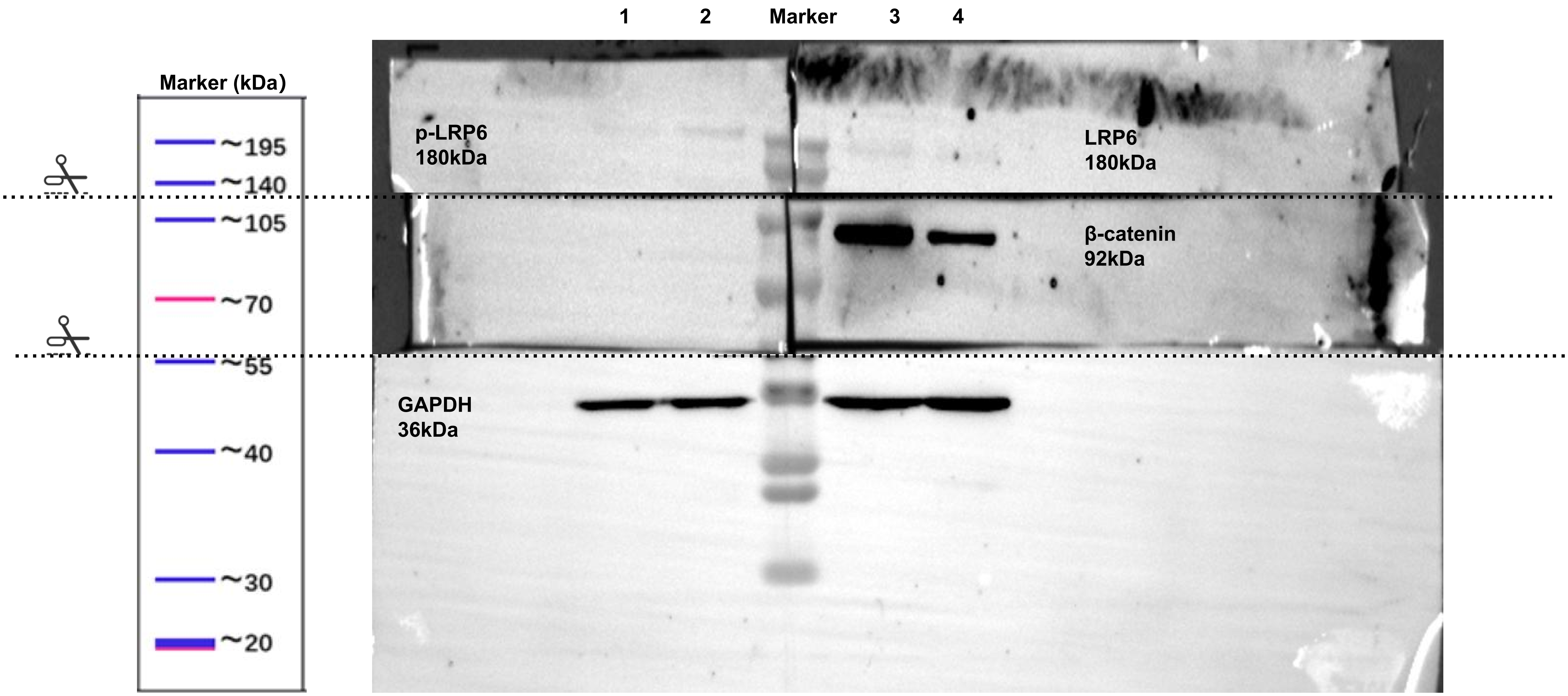

Figure S5. Expression of Wnt signaling-related proteins after interference of BVES

Note: Lanes 1 and 4 are both si-NC; Lanes 2 and 3 are both siRNA (Expose for 30 seconds).
